# Impact of adsorptive CRRT membranes on vancomycin pharmacokinetics in critically ill patients

**DOI:** 10.1080/0886022X.2026.2728756

**Published:** 2026-09-24

**Authors:** Hongxia Zou, Xiang Li, Qing Xu, Zhifeng Zhou, Lu Jin, Fang Wang, Yingying Yang, Ling Zhang

**Affiliations:** ^a^ Department of Nephrology, Kidney Research Institute, West China Hospital of Sichuan University, Chengdu, China; ^b^ Department of Intensive Care Unit, Lhasa People’s Hospital, Lhasa, Tibet, China

**Keywords:** Continuous renal replacement therapy, vancomycin, adsorption, AN69ST, pharmacokinetics, therapeutic drug monitoring

## Abstract

Continuous renal replacement therapy (CRRT) is a vital organ support modality for critically ill patients. Adsorptive membranes, particularly AN69ST and oXiris, are increasingly used in clinical practice; however, their impact on the pharmacokinetics of antibiotics with a narrow target concentration range between efficacy and toxicity, such as vancomycin, remains incompletely defined. Considerable heterogeneity exists among available studies. Multiple in vitro dynamic circulation models reported adsorption of 12% to 19.5% of the initial vancomycin dose, whereas some static or open-model *in vitro* studies failed to detect significant adsorption. Retrospective studies have reported that adsorption accounted for approximately 15.9% with AN69ST membranes and 5.4% with oXiris membranes of total vancomycin elimination. However, the clinical significance of vancomycin removal by adsorptive membranes *via* adsorption remains unclear and warrants further investigation. Nevertheless, overlooking the additional clearance contributed by AN69ST or oXiris membranes during dose regimen design may lead to subtherapeutic vancomycin exposure, thereby increasing the risk of treatment failure and antimicrobial resistance. This article systematically reviews the current evidence on vancomycin elimination characteristics with adsorptive membranes, with an emphasis on adsorption mechanisms, influencing factors, and clinical dosing adjustment strategies. In current clinical practice, intensified therapeutic drug monitoring with dynamic dose adjustment based on measured concentrations remains an essential strategy to ensure the efficacy and safety of vancomycin therapy. A deeper understanding of membrane-specific adsorption kinetics is required to optimize antibiotic dosing in critically ill patients receiving CRRT with highly adsorptive membranes.

## 1. Introduction

Continuous renal replacement therapy (CRRT) continuously removes solutes and fluid from the body through diffusion, convection, and adsorption [1,2]. Owing to its slow and continuous nature, CRRT is particularly suitable for critically ill patients with hemodynamic instability [3]. As a key component of CRRT, membrane materials have evolved from simple solute removal to multifunctional integration. Among the various membrane materials, the AN69 membrane, a copolymer of acrylonitrile and sodium methallylsulfonate, was one of the first synthetic membranes introduced into clinical practice and exhibits adsorptive capacity [4,5]. Other membrane types include polysulfone (PS), polyethersulfone (PES), polyamide (PA), and polymethyl methacrylate (PMMA).

In recent years, adsorptive membranes represented by AN69ST and oXiris have been widely used in clinical practice due to their adsorption properties. Both filters are modifications of the AN69 membrane, with surface modifications enabling functional expansion and optimization. The oXiris membrane integrates four major functions: renal replacement, cytokine removal, endotoxin adsorption, and regional anticoagulation [6–8]. Sepsis and AKI are diagnosed according to the Sepsis-3 consensus and the Kidney Disease: Improving Global Outcomes (KDIGO) criteria, respectively [3,9,10], and sepsis-associated acute kidney injury is defined as acute kidney injury occurring in the setting of sepsis [11]. Approximately 11% of these patients require CRRT support [12]. Pathogen-associated molecular patterns from microorganisms and damage-associated molecular patterns from injured cells are involved in the pathogenesis of sepsis-associated acute kidney injury, making them attractive therapeutic ­targets [13].

Preliminary evidence from several studies suggests that the oXiris membrane may reduce cytokine and endotoxin levels in patients with septic shock, and a meta‑analysis has indicated potential associations with lower 28-day mortality and shorter ICU length of stay [14]. However, the same analysis did not confirm significant differences in 90-day mortality, ICU mortality, or hospital mortality.

In patients undergoing cardiac surgery, a randomized trial reported a reduction in AKI incidence from 39.7% to 28.4% with oXiris [15]. Conversely, a pilot randomized controlled trial (CLEAN ECMO, *n* = 40) in patients with cardiogenic shock on VA-ECMO found that oXiris did not significantly reduce endotoxin levels or improve clinical outcomes, including ECMO weaning success or mortality [16]. Moreover, the 2026 Surviving Sepsis Campaign guidelines do not recommend hemoadsorption or hemoperfusion for sepsis-associated acute kidney injury without specific indications, citing insufficient evidence of benefit [17]. Furthermore, while removing inflammatory mediators, these membranes may also adsorb and eliminate therapeutic drugs, raising concerns about dosing adjustment, particularly for agents that require therapeutic drug monitoring (TDM) for individualized dosing, i.e., those with effective concentrations lying close to toxic concentrations [18–20]. Collectively, these mixed findings underscore the need for a better understanding of drug pharmacokinetics during these extracorporeal therapies to optimize outcomes in this vulnerable population.

Vancomycin is a glycopeptide antibiotic widely applied in the treatment of methicillin-resistant Staphylococcus aureus infections and is commonly used in critically ill patient populations [21,22]. It has a narrow therapeutic window, i.e., a narrow margin between effective and toxic concentrations, and exhibits considerable pharmacokinetic variability, necessitating TDM for dose adjustment [23]. Building upon TDM, target concentration intervention (TCI), as an evolution of this concept, aims to achieve predefined pharmacological targets in individual patients, thereby offering a superior strategy for concentration guided dosing [24,25]. The 2009 consensus on vancomycin TDM recommended a target trough concentration of 10–20 mg/L [26]. The 2020 revised guidelines recommended the ratio of the area under the concentration-time curve to the minimum inhibitory concentration (AUC/MIC) as the monitoring parameter, with a target of 400–600 mg × h/L [27]. Below this target, treatment failure and resistance are more likely, whereas excessive exposure significantly increases the risks of nephrotoxicity and ototoxicity [28]. In critically ill patients, factors such as capillary leakage, fluid resuscitation, and hypoalbuminemia lead to significant alterations in the volume of distribution and clearance of vancomycin, further complicating dose adjustment [29].

The pharmacokinetics of vancomycin during CRRT are influenced by multiple factors, with reported clearance ranging from 0.7 L/h to more than 3.0 L/h and volume of distribution ranging from 0.8 L/kg (IQR 0.6–1.1 L/kg) to more than 100 L/kg [30–32]. This wide variability reflects the differential impact of extracorporeal factors and patient-specific characteristics, as well as the marked heterogeneity among critically ill patients in disease severity and volume status, and further underscores the critical role of therapeutic drug monitoring in this population. Furthermore, studies have shown that CRRT with adsorptive membranes may result in additional vancomycin clearance [33,34]. Even when using guideline-recommended loading and maintenance doses of vancomycin for CRRT, a considerable proportion of patients fail to achieve target drug concentrations [35]. However, due to considerable heterogeneity among existing studies, clinicians lack clear evidence-based guidance when making dose adjustment decisions.

This review aims to systematically review the impact of adsorptive membranes on vancomycin pharmacokinetics, focusing on adsorption characteristics, influencing factors, available evidence, and future research directions.

## 2. Types of adsorptive membrane

### 2.1. AN69/AN69ST membrane

The AN69 membrane, developed by Rhône-Poulenc (France) in 1969, was the first synthetic membrane specifically designed for dialysis. It is a copolymer of acrylonitrile and sodium methallylsulfonate [36], with a hydrophilic hydrogel structure that provides excellent diffusion and water permeability [5]. The sulfonate groups distributed throughout the AN69 structure enable adsorption of inflammatory mediator proteins with isoelectric points above 7 *via* ionic bonding [5,37]. In addition, its symmetric structure facilitates solute diffusion across the membrane matrix.

In the 1990s, hypersensitivity reactions were observed in some patients undergoing hemodialysis with the AN69 membrane, particularly those receiving concomitant angiotensin-converting enzyme inhibitors (ACEIs). These reactions, termed contact phase reactions, were attributed to the strongly negative surface charge (i.e., ζ potential, the electrostatic potential at the membrane liquid interface [38]) of the AN69 membrane, which triggers factor XII activation and activates the kallikrein-kinin system, leading to bradykinin generation. ACEIs interfere with the normal degradation of bradykinin, resulting in its accumulation, which may trigger anaphylactoid reactions and increase anticoagulation requirements [39,40].

To overcome this limitation, researchers developed the AN69ST (Surface Treatment) membrane, which is coated with positively charged polyethyleneimine (PEI) on its surface. This coating reduces the surface ζ potential and attenuates contact phase reactions, thereby decreasing bradykinin generation and improving biocompatibility. PEI modified AN69ST membranes exhibit reduced surface negative charge and enable heparin binding during priming with heparin containing saline. They attenuate factor XII-mediated contact activation and locally mediate antithrombin III-dependent inactivation of factor IIa and Xa at the blood membrane interface. This reduces the requirement for circulating plasma heparin, lowering systemic anticoagulation demands in patients at high bleeding risk and delaying clot-related events within the extracorporeal circuit [41,42].

Meanwhile, the sulfonate groups within the AN69ST membrane matrix preserve its capacity to effectively adsorb and eliminate a broad range of inflammatory mediators, including cytokines (e.g., IL-6 [43,44], TNF-α [44]) and high mobility group box 1 protein [45]. Meanwhile, the sulfonate groups within the AN69ST membrane matrix preserve its capacity to effectively adsorb and eliminate a broad range of inflammatory mediators, including cytokines [46,47].

### 2.2. oXiris membrane

The oXiris membrane represents a technological innovation based on AN69ST. It is treated with a multi-layer linear PEI cationic polymer. The PEI concentration is several times higher than that of AN69ST, resulting in a denser and more stable positive charge layer (Figure 1). This enhances cytokine adsorption and adds endotoxin adsorption capacity [6,7].

**Figure 1.**
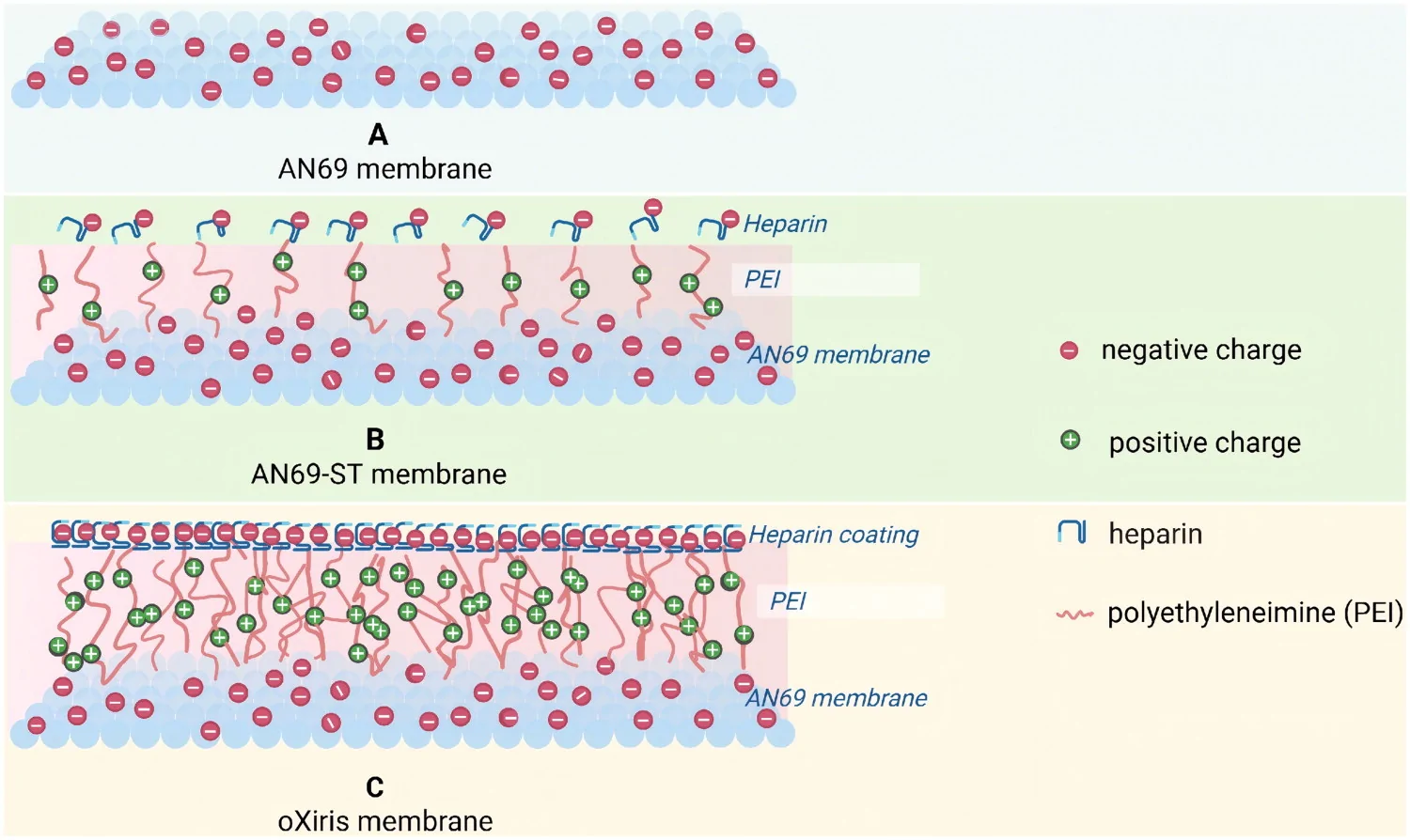
Surface characteristics of AN69 (A), AN69-ST (B), and oXiris membranes (C). Created in BioRender. Zou (2026) https://BioRender.com/h3erm2x.

The AN69 base membrane removes small solutes and water through diffusion and convection, thereby maintaining internal environment stability [48]. The PEI coating alters membrane surface polarity, enhancing its adsorption capacity for cytokines (e.g., IL-6, IL-8, TNF-α) [49–51]. The high-concentration PEI layer provides dense positive charge sites that specifically adsorb negatively charged endotoxin molecules *via* ionic bonding [52]. The pre-grafted heparin coating (4500 IU/m^2^) provides local anticoagulation within the membrane. This significantly reduces or even avoids the use of systemic anticoagulants, thereby lowering bleeding risk [53]. The oXiris membrane attenuates inflammatory injury and improves renal perfusion through the removal of excessive inflammatory mediators, showing potential in improving outcomes of SA-AKI patients [54–56]. Accordingly, the 2024 Asia-Pacific expert consensus recommended the use of the oXiris membrane in septic patients requiring CRRT who present with hemodynamic instability, cytokine storm, severe lactic acidosis, or coagulopathy [57]. However, it is important to note that the 2026 Surviving Sepsis Campaign guidelines did not recommend routine hemoadsorption or hemoperfusion for SA-AKI without specific indications, citing insufficient evidence of benefit [17].

### 2.3. Polymethyl methacrylate (PMMA) membrane

The PMMA membrane also has certain adsorption properties [58]. However, its adsorption capacity differs from that of AN69ST. A study by Uchida et al. showed that the adsorption percentage rate of the PMMA membrane for vancomycin was approximately 4.6%, which is significantly lower than that of AN69ST (12.1%) [59]. Therefore, the PMMA membrane is not discussed in this review.

## 3. Mechanisms of antibiotic elimination during CRRT: underlying principles and influencing factors

### 3.1. Elimination mechanisms

CRRT removes solutes through three basic mechanisms: diffusion, convection, and adsorption (Figure 2) [60].

**Figure 2.**
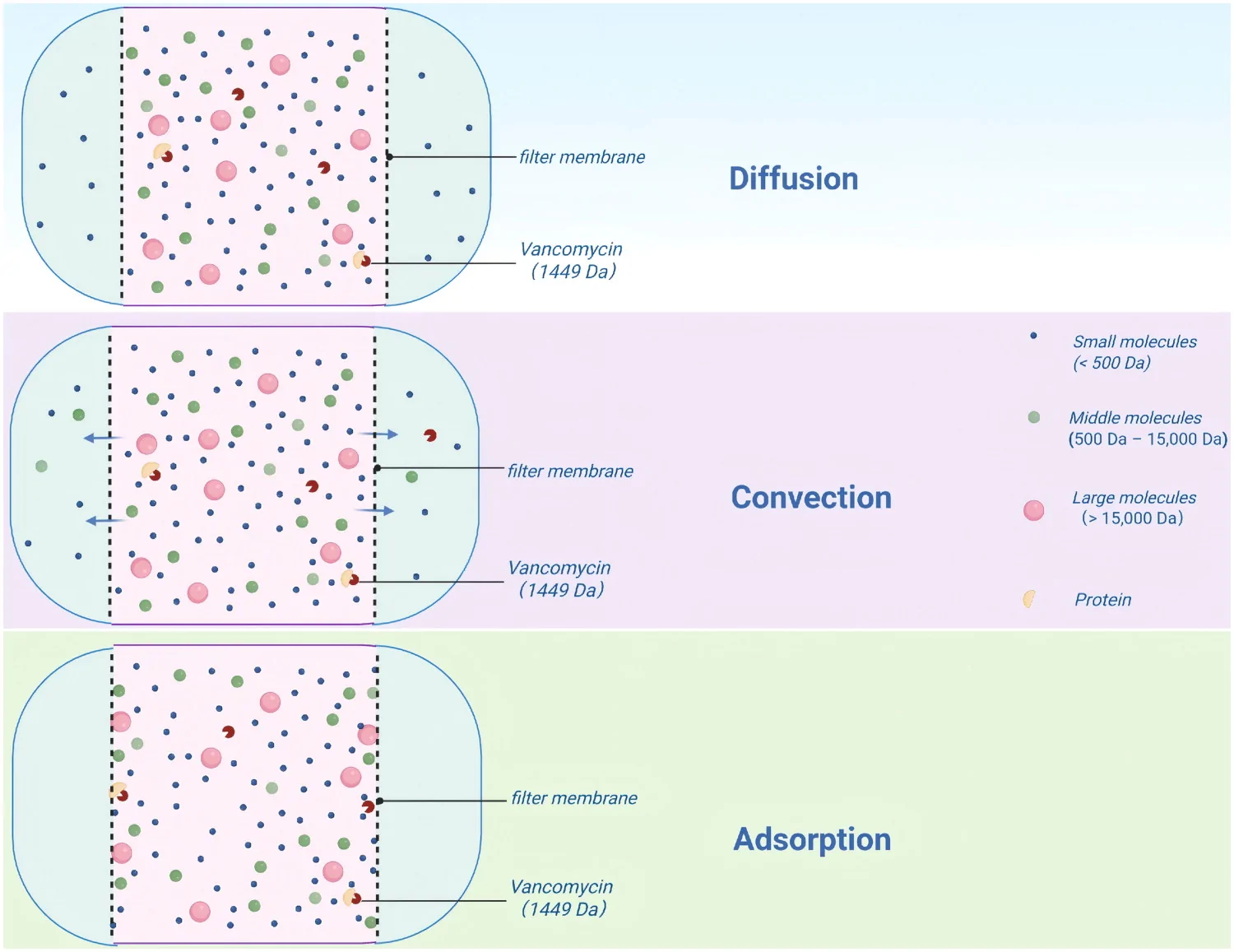
Schematic diagram of three solute clearance mechanisms in CRRT. Created in BioRender. Zou (2026) https://BioRender.com/gh05ji2.

Diffusion is the passive transport of solutes from a high-concentration side to a low-concentration side along a concentration gradient. It primarily depends on solute molecular weight, membrane pore size, dialysate flow rate, and concentration gradient. Diffusion is most efficient for small solutes (molecular weight < 500 Da). Its efficiency decreases exponentially as molecular weight increases. Although diffusion is less efficient at clearing middle molecules such as vancomycin, it does contribute to vancomycin clearance, e.g., in intermittent hemodialysis or CVVHD alone [61].

Convection is the transport of solutes together with water across the membrane. It primarily depends on ultrafiltration rate, sieving coefficient (SC), and membrane characteristics [62]. The sieving coefficient is the ratio of solute concentration in the ultrafiltrate to that in the plasma; a value approaching 1.0 indicates unrestricted passage across the membrane [63]. Convection is effective for both small and middle molecular weight solutes, and those with a sieving coefficient close to 1.0 can be efficiently removed by this mechanism.

Adsorption is the process by which solutes attach to the filter membrane surface through electrostatic interactions, hydrophobic interactions, or van der Waals forces. It does not depend on the concentration gradient or transmembrane pressure. Instead, adsorption depends on membrane surface properties and the physicochemical characteristics of the drug.

*In vitro* studies have suggested that polyacrylonitrile (PAN) membranes may exhibit adsorption effects on various antibiotics [64–67]. Differences in the physicochemical properties of antibiotics appear to contribute to marked variations in their adsorption propensity. Vancomycin, owing to its moderate molecular weight and positive charge, may be among the antibiotics whose elimination is notably influenced by adsorption [68]. Onichimowski et al. reported a net vancomycin adsorption of 195.26 mg (19.5% of the total dose) on an AN69ST filter in a whole-blood model over a 2-h CRRT session [33]; under identical conditions in a crystalloid model, the same group observed an adsorption of 181.88 mg (18.2% of the total dose) [34]. Tian et al. demonstrated that a 0.6 m^2^ AN69 filter adsorbed approximately 10.08 mg of vancomycin, corresponding to 28% of the administered dose, within 2 h in a blood‑crystalloid mixture [69]. These findings suggest that membrane adsorption may contribute to vancomycin elimination during CRRT, although the absolute amount appears to vary considerably depending on experimental conditions, drug concentration, and membrane type. In light of these observations, the potential impact of adsorption on drug dosing should be carefully considered when individualizing antibiotic therapy in clinical practice [70].

### 3.2. Influencing factors

Antibiotic elimination during CRRT is influenced by multiple factors related to filter characteristics, drug physicochemical properties, and patient status [20,65,71,72] (Figure 3).

**Figure 3.**
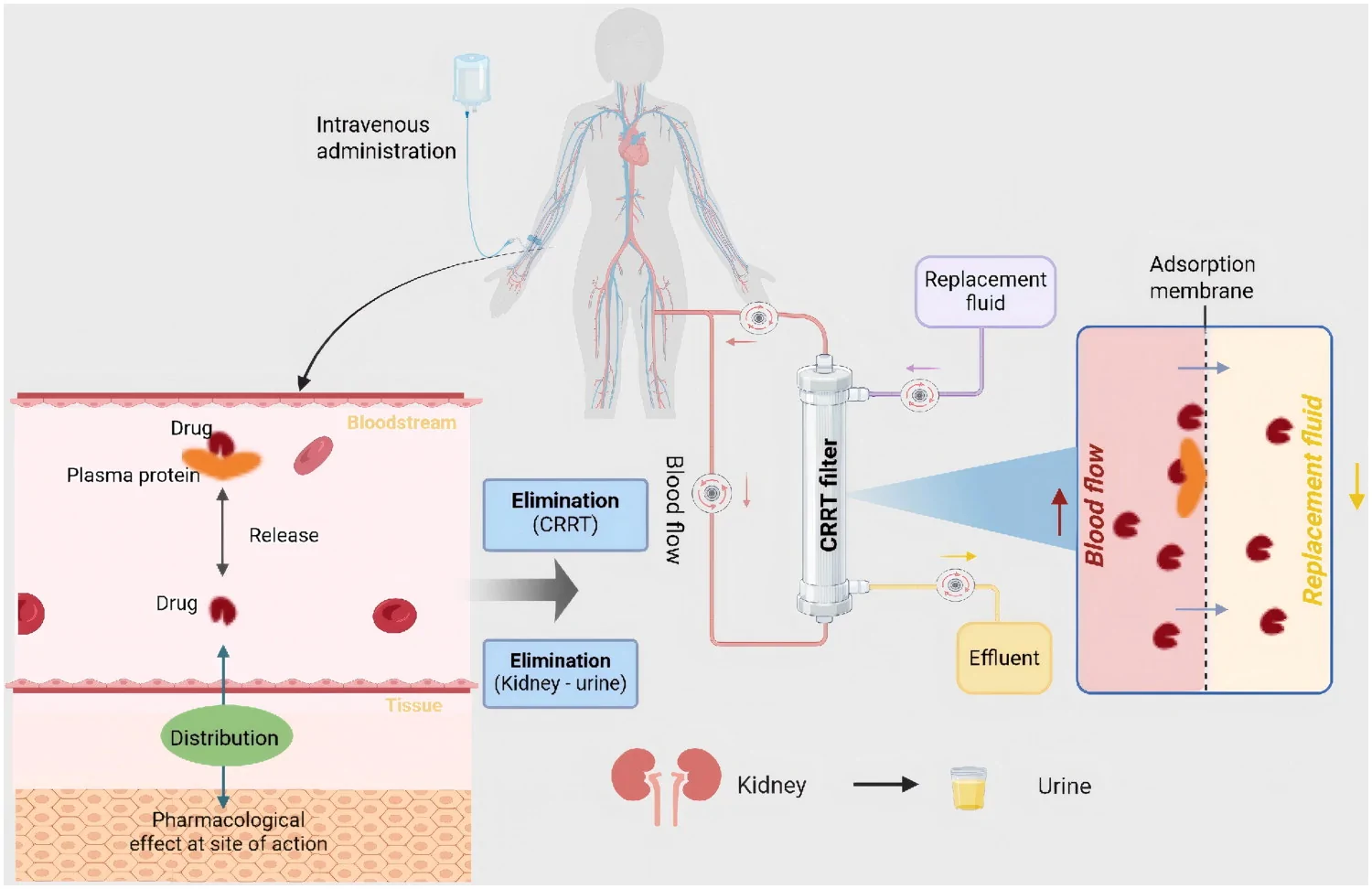
Schematic diagram of vancomycin pharmacokinetics during CRRT. Created in BioRender. Zou (2026) https://BioRender.com/wrd48dh.

#### 3.2.1. Drug factors

Molecular weight directly affects the elimination pathway. Small-molecule drugs are cleared predominantly by diffusion and convection, whereas middle- to large-molecule drugs rely primarily on convection and adsorption. The molecular weights of commonly used antibiotics range from 240 to 1748 Da.

Protein binding rate is a key factor. Drugs with high protein binding rates are not easily cleared by CRRT because only unbound drugs can pass through membrane pores [2]. Studies have shown that the unbound drug fraction is moderately correlated with the sieving coefficient (R^2^ = 0.55) and can serve as a surrogate marker for predicting CRRT elimination efficiency [73]. Most unbound drugs can be cleared by CRRT [74].

Charge properties are also critical. Positively charged drugs are easily adsorbed by negatively charged membrane materials through electrostatic interactions. Conversely, negatively charged drugs may be adsorbed by positively charged membrane materials. The degree of charge matching between the drug and the membrane surface affects adsorption efficiency.

Volume of distribution (Vd) is the ratio between the total amount of drug in the body and the plasma concentration [75]. Volume of distribution does not directly affect elimination; rather, it determines the relationship between the amount of drug in the body and the plasma concentration. Drugs with a large Vd are extensively distributed into tissues, resulting in low plasma concentrations relative to the total body drug amount; consequently, a given clearance value removes a smaller fraction of the total body drug per unit time, prolonging the elimination half-life [76]. In contrast, drugs with a small Vd and low protein binding are mainly distributed in the plasma and extracellular fluid, making them more susceptible to elimination by CRRT.

For many drugs primarily eliminated renally, elimination increases significantly during CRRT, and dose adjustment is required. For drugs with renal elimination below 25–30% that are primarily eliminated through non-renal pathways, CRRT contributes little to total elimination, and dose adjustment is generally not required. However, it should be noted that fluid removal by CRRT may also moderately increase the elimination of drugs eliminated by other organs.

#### 3.2.2. CRRT factors

Antibiotic elimination during CRRT is differentially affected by prescribed CRRT intensity, modality, membrane type, and the actual delivered dose, the latter often being lower than prescribed due to circuit downtime and filter clotting [77–79]. CRRT intensity is commonly defined by the prescribed effluent flow rate, which comprises both ultrafiltration and dialysate flow rates. Higher ultrafiltration rates increase convective clearance [80,81]. Higher dialysate flow rates increase diffusive clearance [82]. Regarding treatment modality, the relative drug clearance efficiency among CVVH, CVVHD, and CVVHDF is primarily determined by drug-specific characteristics and treatment parameters. At equivalent total effluent flow rates, the differences in drug elimination among these modalities are generally clinically negligible for most drugs [83].

Adsorption capacity differs significantly among membrane materials. AN69 series membranes have the highest adsorption capacity, followed by PMMA, while PS and PES membranes have the weakest adsorption capacity [61]. Filter runtime is an important factor. Adsorption becomes gradually saturated over time. New filters have the strongest adsorption capacity during the initial period. Enhanced drug concentration monitoring is required after filter change [68,78,84]. A broad range of CRRT filter surface areas, typically ranging from 0.6 to 1.5 m^2^ or larger, are now available to accommodate different patient sizes and treatment requirements [65]. Filter surface area is an important determinant of drug clearance, with larger filters generally providing higher clearance for a given treatment modality. Studies have shown that patients receiving 1.5 m^2^ AN69ST filters require higher antibiotic doses compared with those receiving 0.9 m^2^ filters [77]. Membrane surface charge is also critical. Negatively charged membranes more readily adsorb positively charged drugs.

#### 3.2.3. Patient factors

Critically ill patients exhibit substantial interindividual variability, which differentially affects antibiotic pharmacokinetics. Residual renal function may increase drug elimination, while drug elimination in anuric patients depends exclusively on extrarenal pathways, including metabolism, hepatic elimination, and CRRT [85]. Volume status affects volume of distribution. For example, volume overload increases volume of distribution, leading to reduced plasma drug concentrations and therefore decreased presentation of drug to the filter per unit time. Albumin level affects the protein binding rate of drugs. Hypoalbuminemia increases the proportion of unbound drug, potentially enhancing drug elimination.

Sepsis induces complex pathophysiological changes. Inflammatory responses increase capillary permeability, accompanied by fluid accumulation and hypoalbuminemia. These changes increase the volume of distribution, prolong antibiotic half-life, alter protein binding rates of various antibiotics, and significantly affect drug metabolism [73]. In addition, liver and kidney function may be impaired in septic patients, further interfering with drug elimination. These interacting factors make antibiotic pharmacokinetics highly unpredictable in septic patients, reinforcing the need for intensified TDM as an essential strategy [86].

## 4. Vancomycin elimination by adsorptive filters

### 4.1. In vitro evidence

Vancomycin has a molecular weight of 1,449 Da and a protein binding rate of approximately 30–55%. It is primarily excreted *via* the kidneys and carries a positive charge under physiological pH conditions [87]. *In vitro* evidence of vancomycin adsorption by adsorptive filters is shown in Table 1.

**Table 1.** Summary of *in vitro* studies on vancomycin adsorption by adsorptive filters.

Study	Experimental model	Filter type	Key findings	Adsorption percentage (%)^a^
Quale et al. [91]	Clinical observation + *in vitro* experiment	PAN membrane	Serum vancomycin concentration decreased by ∼37% after hemodialysis; adsorption was independent of plasma proteins.	Not quantified
Tian et al. [70]	Blood-crystalloid mixture, CVVH mode, 120 min	AN69 (0.6 m²)	AN69 adsorbed significantly more than polyamide and polysulfone; ∼1/3 of the total drug was adsorbed within 2 h; irreversible	∼33%
Shiraishi et al. [92]	Static shaking incubation, no transmembrane pressure, no convection	PAN membrane	No vancomycin adsorption observed	0%
Onichimowski et al. [34]	Crystalloid circulation model, 120 min	AN69ST (1.5 m²)	Concentration dropped to 44.5% of the initial at 5 min; membrane adsorption accounted for 18.2% at 120 min; desorption after saturation	18.2%
Onichimowski et al. [33]	Whole-blood model (porcine blood), 120 min	AN69ST (1.5 m²)	Net adsorption 195.26 mg (19.5% of total); minimal increase vs. crystalloid model	19.5%
Uchida et al. [60]	CHDF circuit model; varying albumin concentrations (0–5.0 g/dL) and effluent flow rates (800–3000 mL/h)	AN69ST	Diffusion/convection contribution: 82.1 ± 9.8%; adsorption percentage: 12.1 ± 6.1%; adsorption decreased with higher albumin	12.1 ± 6.1%
Baud et al. [93]	Open model (NeckEpur technology); CDF and CD modes	ST150^®^	No adsorption detected	0%

PAN: polyacrylonitrile; AN69ST: AN69 surface-treated membrane; ST150^®^: ST150^®^ filter; CHDF: continuous hemodiafiltration; CVVH: continuous venovenous hemofiltration; CDF: continuous diafiltration; CD: continuous dialysis.

^a^Adsorption percentage is defined as the percentage of the total vancomycin amount removed by membrane adsorption during the experimental period, calculated as: Adsorption percentage (%) = (amount of vancomycin adsorbed onto the membrane/initial vancomycin dose) ×100.

As early as 1992, Quale et al. demonstrated vancomycin adsorption by PAN membranes through clinical observations and *in vitro* experiments. After hemodialysis with a PAN membrane, serum vancomycin concentrations decreased by approximately 37%, and this adsorption was independent of plasma proteins [88]. Subsequently, Tian et al. conducted an *in vitro* study in which 36 mg of vancomycin was added to a blood–crystalloid mixture. Drug concentration changes were monitored over 120 min under CVVH mode. The results showed that the AN69 membrane adsorbed significantly more vancomycin than polyamide and polysulfone membranes. A 0.6 m^2^ AN69 filter adsorbed approximately one-third of the total vancomycin within 2 h, and the adsorption was irreversible [69].

In 2020, Onichimowski et al. conducted the first series of *in vitro* studies on vancomycin adsorption using a 1.5 m^2^ AN69ST filter. Using a closed-loop single-compartment crystalloid model containing 1000 mg vancomycin in 5 L of buffered crystalloid fluid, the concentration declined to 44.5% of baseline within 5 min, and 181.88 mg (18.2%) was adsorbed onto the membrane by 120 min [34]. The dynamic changes in adsorption suggested that as adsorption sites on the AN69ST filter gradually became saturated, previously adsorbed vancomycin began to desorb. Subsequently, the research team used a closed-loop whole-blood model with 1000 mg of vancomycin added to 1010 mL of porcine blood, yielding an initial concentration of approximately 990 mg/L. After 2 h of circulation, the vancomycin adsorption was 195.26 mg, representing 19.5% of the total amount [33]. Compared with the crystalloid model, the adsorption amount in the whole-blood model increased only slightly, indicating that the presence of plasma proteins had a minimal impact on adsorption. Notably, the initial vancomycin concentrations used in these two *in vitro* experiments, 200 and 990 mg/L, are considerably higher than those typically observed within the therapeutic window. The adsorption percentage tends to decrease with increasing vancomycin doses, as membrane binding capacity is finite and binding sites may become progressively saturated. Therefore, the adsorption percentages observed under these experimental conditions may not be directly applicable to clinical practice.

Uchida et al. used an *in vitro* continuous hemodiafiltration (CHDF) circuit model with an initial vancomycin concentration of 50 mg/L. The study was conducted under different human serum albumin concentrations (0, 2.5, and 5.0 g/dL) and effluent flow rates (800, 1500, and 3000 mL/h). The total vancomycin clearance (CL_CHDF_) with the AN69ST membrane increased with higher effluent flow rates and decreased with higher albumin concentrations. Specifically, at an effluent flow rate of 800, 1,500, and 3,000 mL/h, CL_CHDF_ values were 0.76 ± 0.06L/h, 1.47 ± 0.03L/h, and 2.63 ± 0.19 L/h, respectively, in the absence of albumin; at an albumin concentration of 5.0 g/dL, the corresponding values were 0.67 ± 0.03L/h, 1.30 ± 0.02L/h, and 2.18 ± 0.04 L/h. Across all conditions, the diffusion/convection contribution rate was 82.1 ± 9.8%, and the adsorption percentage rate was 12.1 ± 6.1% [59]. Although adsorption was not the dominant clearance mechanism, its contribution of approximately 12% should not be overlooked.

However, not all *in vitro* studies have observed consistent vancomycin adsorption. When experimental conditions were altered, the adsorption effect sometimes disappeared. Shiraishi et al. used a static shaking incubation model without transmembrane pressure or convection and observed no vancomycin adsorption by the PAN membrane [89]. Baud et al. conducted a study using an open *in vitro* model based on NeckEpur technology. Vancomycin was added to a central reservoir simulating human systemic circulation, yielding a mean initial concentration of 51.4 ± 5.0 mg/L. Simulated blood circulated through an ST150 filter, and the resulting effluent was completely collected without recirculation into the system. Two clinically common post-dilution CRRT modalities were tested: continuous diafiltration (CDF) and continuous dialysis (CD). The mean clearances derived from central-chamber concentration-time data were 2.8 L/h for CDF and 1.9 L/h for CD, whereas the mean clearances calculated from cumulative effluent drug recovery were 4.4 L/h and 2.2 L/h, respectively. Of note, effluent recovery (114% for CDF and 108% for CD) exceeded central chamber elimination (91% for both modalities), suggesting that significant filter adsorption is unlikely [90]. Given the inherent limitations of *in vitro* experiments, these findings should be extrapolated to clinical CRRT practice with great caution.

### 4.2. In vivo evidence

Kirwan et al. conducted a retrospective study including 24 critically ill patients receiving CVVHDF with a non-adsorptive PAES membrane. Probability of target attainment simulations indicated that a vancomycin regimen consisting of a 2 g loading dose followed by 750 mg every 12 h as a maintenance dose enabled most patients to achieve an early trough concentration above 15 mg/L [91].

The 2020 guidelines from the American Society of Health-System Pharmacists and the Infectious Diseases Society of America recommend an initial vancomycin maintenance dose of 7.5–10.0 mg/kg every 12 h when the CRRT dose is 20–25 mL/kg/h [92]. The 2024 Chinese expert consensus on antibiotic dose adjustment during CRRT recommends a vancomycin loading dose of 20 mg/kg and a maintenance dose of 500 mg every 12 h in CVVHDF mode [74]. However, these recommended doses are primarily based on studies using non-adsorptive filters (e.g., polysulfone, polyethersulfone) and do not fully account for the impact of highly adsorptive filters such as AN69ST or oXiris.

Honore et al. pointed out that in current clinical practice, over 95% of Prismaflex treatments use the adsorptive AN69ST filter. Continuing to follow conventional dosing regimens may lead to treatment failure. The team suggested that patients using AN69ST filters require a vancomycin maintenance dose of approximately 3000 mg/day during the first three days of treatment to achieve target concentrations, several times higher than the conventional recommended doses [70].

Tsuruyama et al. conducted a retrospective study including 10 patients using AN69ST filters [93]. Total vancomycin clearance (CL_total_) was calculated using a one-compartment model with the Sawchuk-Zaske method, yielding a value of 1.84 L/h (95% CI: 1.38–2.30 L/h). The AN69ST filter clearance (CL_CHDF_) was determined by measuring the inlet-to-outlet concentration gradient across the filter, using the formula CL_CHDF_ = [(Q_Bin_ × C_Bin_) − (Q_Bout_ × C_Bout_)]/C_Bin_, and was estimated to be 0.69 L/h (95% CI: 0.52–0.86 L/h). The theoretical clearance (CL_theol_), which represents clearance based on diffusion and convection alone without accounting for adsorption, was calculated from the waste liquid flow rate and was 0.58 L/h [80]. The difference between CL_CHDF_ (0.69 L/h) and CL_theol_ (0.58 L/h) was approximately 0.11 L/h, corresponding to an adsorption percentage of approximately 15.9% [80]. To maintain a target trough concentration of 15 μg/mL, the estimated average 24-h dose was approximately 934.6 mg.

A recently published retrospective study was the first to include the oXiris filter as a covariate in a population pharmacokinetic model of vancomycin. The study included 55 patients with 126 vancomycin concentration measurements. The results showed that use of the oXiris membrane was associated with a 5.4% increase in vancomycin clearance [84]. This finding may be attributed to competitive adsorption of inflammatory mediators by oXiris and adsorption sites that approach saturation within 6–8 h. Given that trough concentration sampling is performed after the peak adsorption period has passed, the clinical impact is likely to be limited.

Currently, studies on vancomycin pharmacokinetics with the oXiris membrane are very limited. With the development and application of population pharmacokinetic modeling software such as Monolix [94,95] and integrated dialysis pharmacokinetic models such as IDP [96] and integrated dialysis pharmacokinetic models such as IDP [19], a search of ClinicalTrials.gov and the Chinese Clinical Trial Registry (ChiCTR) identified three ongoing studies (NCT06543940, NCT06261164, ChiCTR2600120094). Their results will provide important evidence for vancomycin dose adjustment when using oXiris filters.

## 5. Discussion

The adsorption of vancomycin can be attributed to the interaction between the physicochemical properties of the drug and the membrane surface characteristics. Vancomycin has a moderate molecular weight and carries a positive charge under physiological pH conditions. The AN69 membrane is a copolymer of acrylonitrile and sodium methallylsulfonate. Its sulfonate groups confer a strong negative charge on the membrane surface, enabling the adsorption of positively charged vancomycin molecules. Quale et al. observed that changing the pH of the solution directly affected the charge states of both vancomycin molecules and the membrane surface, thereby altering adsorption intensity. This finding confirmed that electrostatic interaction is likely the primary mechanism of adsorption [88]. The study by Inano et al. showed that the vancomycin residual rate was 44.5% at 20 min, with only minimal additional adsorption observed when the duration was extended to 120 min [68]. The adsorption process exhibits saturation characteristics, indicating a limited number of binding sites on the membrane surface.

Synthesizing the available *in vitro* evidence, vancomycin adsorption by AN69 series filters is not constant but depends on multiple factors, including convection intensity, experimental medium, drug concentration, and calculation method. The reported adsorption percentage rates across different studies range from 0% to 19.2% [33,34,59,89,90]. This variability primarily stems from heterogeneity in experimental conditions.

Although some studies using open models that more closely mimic clinical conditions did not observe clinically significant adsorption, most dynamic circulation models have demonstrated the existence of adsorption and its potential contribution to total vancomycin clearance. Tsuruyama et al. estimated that a vancomycin dose of approximately 935 mg/day would be sufficient to maintain a trough concentration of 15 μg/mL when CRRT is performed with non-adsorptive filters [93]. In contrast, Honore et al. suggested that patients using AN69ST filters require a vancomycin maintenance dose of nearly 3000 mg/day during the first three days of treatment [70]. This represents a substantial difference in dosing recommendations.

When using adsorptive filters for CRRT in clinical practice, the additional contribution of adsorption to vancomycin clearance should not be completely ignored [34,59]. Adherence to conventional dosing regimens based on non-adsorptive filters (e.g., polysulfone, polyethersulfone) may result in subtherapeutic dosing [44,62], increasing the risk of treatment failure and antimicrobial resistance. However, the surface modification of AN69ST and oXiris filters reduces their surface negative charge, which may attenuate electrostatic adsorption of vancomycin [97]. Therefore, there is no need for excessive concern that would lead to uniformly adopting extremely high doses. Furthermore, drug metabolism during CRRT is influenced by multiple complex factors. Standard vancomycin dosing regimens often fail to achieve therapeutic levels in critically ill patients receiving renal replacement therapy [98]. Therefore, therapeutic drug monitoring to guide individualized dosing is essential [99].

The studies included in this review have notable methodological heterogeneity. Significant differences exist among studies in terms of experimental models, CRRT modes, treatment parameters, and methods for calculating adsorption percentage, leading to considerable variability in results. Most studies have focused only on AN69 or AN69ST filters, with a lack of research on oXiris filters. Existing *in vivo* studies are predominantly small-sample, single-center, retrospective designs with risks of selection bias and inadequate confounding control. Moreover, current research has primarily focused on adsorption elimination during the initial phase of filter use. Whether drug desorption occurs after adsorption saturation, the rate of such desorption, and its clinical implications remain unclear.

Based on the above limitations, future research should consider the following directions. First, well-designed *in vitro* experiments should quantitatively assess the dynamic changes in adsorption percentage under different convection intensities. Second, prospective studies should be conducted to develop population pharmacokinetic models of vancomycin specifically tailored to adsorptive filters, incorporating covariates such as filter type, treatment parameters, and patient characteristics to enable individualized dose prediction. Third, the scope of research should be extended to other antibiotics susceptible to adsorption, ultimately leading to the development of antibiotic class-specific dosing guidelines for the use of adsorptive filters.

## 6. Conclusion

Current clinical evidence is primarily derived from *in vitro* and small-sample retrospective studies. Substantial disagreement exists regarding the extent of additional vancomycin elimination by adsorptive filters under different experimental conditions. Existing guidelines for vancomycin dosing during CRRT have not incorporated the impact of filter type. Therapeutic drug monitoring is essential. Large-scale prospective studies are urgently needed to establish precise dosing strategies.

## Data Availability

Data sharing is not applicable to this article as no new data were created or analyzed in this study.
